# Comprehensive evaluation of NT5E/CD73 expression and its prognostic significance in distinct types of cancers

**DOI:** 10.1186/s12885-018-4073-7

**Published:** 2018-03-07

**Authors:** Tao Jiang, Xiaofeng Xu, Meng Qiao, Xuefei Li, Chao Zhao, Fei Zhou, Guanghui Gao, Fengying Wu, Xiaoxia Chen, Chunxia Su, Shengxiang Ren, Changyun Zhai, Caicun Zhou

**Affiliations:** 1 Department of Medical Oncology, Shanghai Pulmonary Hospital, Thoracic Cancer Institute, Tongji University School of Medicine, No. 507, Zheng Min Road, Shanghai, 200433 People’s Republic of China; 2 Department of Clinical Laboratory, Fudan University Shanghai Cancer Center, Shanghai, 200032 People’s Republic of China; 3 Department of Oncology, Shanghai Medical College, Fudan University, Shanghai, 200032 People’s Republic of China; 4 Department of Lung Cancer and Immunology, Shanghai Pulmonary Hospital, Thoracic Cancer Institute, Tongji University School of Medicine, Shanghai, 200433 People’s Republic of China; 5 Department of Medical Oncology, Yancheng TCM Hospital Affiliated to Nanjing University of Chinese Medicine, Yancheng, 224001 People’s Republic of China

**Keywords:** CD73, Cancer, Immunotherapy, Prognosis, Characterization, Meta-analysis

## Abstract

**Background:**

CD73 is one of the critical component in the formation of immunosuppressive microenvironment in cancers. We aimed to provide an overview of the current status of CD73 expression and its relationship with clinicopathlogical features and prognosis in different cancers.

**Methods:**

PubMed, Web of Science, EMBASE and Cochrane library were searched to identify the relevant studies. CD73 expression level in distinct cancers and its relationship with clinicopathlogical characteristics and prognosis were investigated using online database. Meta-analyses were conducted using RevMan v5.0 and STATA v12.0.

**Results:**

Fourteen publications with 2951 cases were included. The incidence of high CD73 expression was 0.50 (95% CI: 0.36–0.63). Data from Oncomine validated that median CD73 expression level in tumor tissues was markedly higher than that in normal tissues in most kinds of cancers except cecum adenocarcinoma and ovarian cancer (*P* < 0.05). High CD73 expression was significantly correlated with shorter overall survival (OS) in various cancers (high risk [HR] = 1.48; *P* < 0.05). Subgroup analysis using online database demonstrated that high CD73 expression was significantly correlated with poor OS in breast (HR = 1.23; *P* < 0.05) and ovarian cancer (HR = 1.14; *P* < 0.05), but favorable OS in lung (HR = 0.80; *P* < 0.05) and gastric cancer (HR = 0.71; *P* < 0.05). High CD73 expression was dramatically associated with lymph node metastases (OR = 2.61; *P* = 0.05).

**Conclusion:**

High CD73 expression was significantly associated with lymph node metastases and a promising prognostic factor in different types of cancers.

**Electronic supplementary material:**

The online version of this article (10.1186/s12885-018-4073-7) contains supplementary material, which is available to authorized users.

## Background

CD73, also designated ecto-5′-nucleotidase (NT5E), is one kind of ecto-nucleotidase that plays a critical role in the catabolism of extracellular ATP to adenosine and the maintenance of immune homeostasis [1, 2]. CD73 is the rate-limiting enzyme in the ATP to adenosine degradation pathway. It can dephosphorylate adenosine monophosphate (AMP) to form adenosine and activate specific G-protein coupled receptor (GPCR) to increase intracellular cAMP level, thus promoting cancer cell aggressiveness, metastasis and angiogenesis [3–7]. Previous studies unraveled that extracellular adenosine concentration was elevated in the tumor microenvironment [8]. Recently, CD73-adensine was found to be a significant pathway involved in the formation of immunosuppressive microenvironment in distinct tumors [3].

CD73-derived adenosine mainly mediates immunosuppression via activation of A2A receptor on immune cells, especially natural killer (NK) cells and CD8+ T cells. Recent studies revealed that CD73 plays a pivotal role in tumor escape from immune surveillance. The mechanism can be summarized into three aspects: (i) inhibition of clonal expansion, activation and homing to tumor specific T cells; (ii) to increase a substantial component of the suppressive capabilities of regulatory T cells (Tregs) and Th17 cells; (iii) to accelerate the conversion of anti-tumor type 1 macrophages into pro-tumor type 2 macrophages [9]. Targeting CD73 results in favorable antitumor effects in preclinical studies and combination of CD73 blockade with other immune checkpoint inhibitors, such as anti-cytotoxic T-lymphocyte antigen (CTLA)-4 antibody or anti-programmed cell death protein (PD)-1/PD-1 ligand (PDL1) antibody, is particularly promising [9]. Increasing evidence suggested that CD73 highly expressed in a wide range of cancer types, including breast cancer, colorectal cancer, glioblastoma, melanoma, prostate cancer, ovarian cancer, and non-small-cell lung cancer (NSCLC). High CD73 expression was often associated with poor prognosis in different cancers. However, several studies demonstrated that high CD73 expression was not correlated with the prognosis of patients with breast cancer [10]. Even some studies indicated that high CD73 expression was associated with favorable prognosis in patients with gastric cancer or rectal adenocarcinoma [11, 12].

To date, there is no study to comprehensively investigate the correlation between high CD73 expression and prognosis in cancer patients. There is also no study to dissect the CD73 expression level in different cancers and the relationship between high CD73 expression and clinicopathlogical characteristics. Herein, we conducted this study with published data and online database to clarify the influence of high CD73 expression and its impact on the outcomes of different cancers, as well as its relationship with clinicopathlogical features. Furthermore, we performed subgroup analysis on the association of high CD73 expression with prognosis in breast, lung, gastric and ovarian cancer by using the published data on KM plotter (http://www.kmplot.com). We aimed to provide an overview of the current status of high CD73 expression in tumor prognosis and future immunotherapy.

## Methods

### Online search

We carried out a publication search through PubMed/Medline, EMBASE, Google Scholar, Cochrane Library and Web of Science until January 31, 2017 (records in English or Chinese). The following keywords was utilized: (“CD73” OR “NT5E” OR “ecto-5′-nucleotidase”) and (“cancer” OR “tumor” OR “carcinoma” OR “neoplasm”). We firstly reviewed the titles and abstracts to determine publications, which investigated the relationship of CD73 expression with overall survival (OS), recurrence free survival (RFS) and clinicopathological characteristics. Reference in each articles were hand-searched. This analysis was conducted in line with Preferred Reporting Items for Systematic Reviews and Meta-Analyses: the PRISMA Statement [13].

### Publication selection

Publications met the following criteria were eligible: (1) investigated high CD73 expression in kinds of human solid tumors; (2) CD73 expression was determined on tumor specimens, instead of the peripheral blood or cell lines or any other types of tissue; (3) reported data could analyze the rate of high CD73 expression and/or high risk (HR) on clinical outcomes. Studies were ineligible if they were: (1) comment, reviews, case-only studies, editorial, or familial studies; (2) insufficient data for analysis of rate and/or HR with 95% confidence intervals (CIs); and (3) repeat of previous publications or replicated samples. The study eligibility was independently evaluated by two reviewers. Disagreements were resolved after discussion with third reviewer.

### Data extraction and quality assessment

We extracted the following information from the included studies: name of first author, publication year, tumor types, study population, high CD73 expression test techniques, cut-off value, and rate of high CD73 expression with 95% CIs, HR for DFS, RFS, and/or OS with related 95% CIs. If the HRs and CIs were not reported, the total death events and the numbers of study population in each group were extracted to indirectly analyze HRs and CIs. To avoid the selection bias, we did not extracted data from the reported Kaplan-Meier curves. When univariate and multivariate analysis were simultaneously reported, the results of multivariate analysis were selected. Two reviewers independently extracted the data by using a predefined Excel form. Disagreements were solved by consensus. As we previously mentioned [14], two reviewers assessed the study quality independently by using the listed factors. Studies lacking any of these criteria would also be excluded.

### Online database cross-validation

To determine the expression level of CD73 in a broader set of cancers and matched normal tissues, we queried the Oncomine, a web-based microarray database (http://www.oncomine.org), to analyze the gene expression level of CD73 in more than 20 types of cancers with distinct histology. We then examined the association of high CD73 expression with prognosis in breast, lung, gastric and ovarian cancer by using the published data on http://www.kmplot.com.

### Statistical analysis

The incidence of high CD73 expression were combined. Respective 95% CIs were determined per estimate and presented in forest plots. For time-to-event data, the HRs with related 95% CIs were directly extracted from the eligible publications or calculated using previous methods proposed by Tierney et al. [15]. Cochran’s Q test and I^2^ statistic were used to determine the heterogeneity of different studies. Low-level heterogeneity was defined as *P* > 0.1 for the χ^2^ test and I^2^ < 25%. If the heterogeneity was non-significant, a pooled effect was calculated with a fixed-effects model. A random-effects model was used when the heterogeneity was statistically significant. Publication bias was assessed by using funnel plots, Begg’s and Egger’s tests. Statistical analysis was conducted by Review Manager 5.0 software and STATA v12.0 (Stata Corporation, TX). All data were analyzed using the Statistical Package for Social Sciences (SPSS) software (version 20.0 for Windows). *P* values were two-sided and considered significant if less than 0.05 except for the Q-test.

## Results

### Characteristics of included studies

**Fig. 1:**
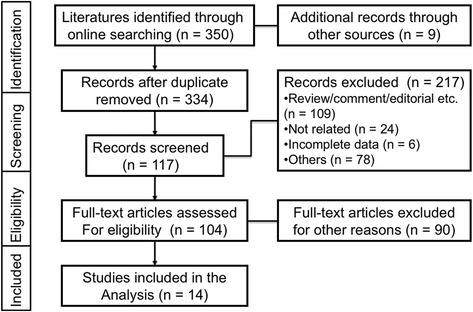
Flowchart of the study inclusion

The result of studies inclusion was listed in Fig. 1. Briefly, a total of 359 potentially relevant publications were found, and 14 studies were finally included in this study after screening [10–12, 16–26]. Most of the excluded abstracts were reviews, comment or studies with incomplete data. In the current analysis, 2951 cases from 14 studies were applied to explore high CD73 expression in 12 types of human cancers. Three studies were in CRC, 2 studies were in ovarian cancer and other 9 studies were about breast, digestive, gynecological, urinary and lung cancer. The main characteristics of the included studies were shown in Table 1. In addition, prognostic data were obtained from all of included studies on OS and 4 of 14 studies on RFS.

**Table 1:** Baseline Characteristics of included studies

Author	Tumor type	Year	No. of cases	CD73+ No.	Positive rate	Test methods	Definition of high expression
Hoon et al.	Ovarian cancer	2012	167	117	70.10%	IHC	using a 4-value grade ( >0 score)*
Anna et al.	Breast cancer	2012	136	101	74.30%	IHC	> median expression levels
Wu et al.	Colorectal Cancer	2012	223	100	44.80%	IHC	using X-tile program to determine the cutoff value
Wu et al.	Colorectal Cancer	2012	135	68	50.40%	IHC	using X-tile program to determine the cutoff value
Lu et al.	Gastric cancer	2013	68	31	45.60%	IHC	semi-quantitative method >5**
Xiong et al.	Gallbladder cancer	2014	108	59	54.60%	IHC	the percent of positively stained cells was >10 %
Martin et al.	Ovarian cancer	2015	208	104	50.00%	IHC	highest 20% CD73 expression was used as a cutoff
Marian et al.	Bladder Cancer	2015	174	46	26.40%	IHC	semi-quantitative 3-scale scoring system: =2***
Yu et al.	Renal cell carcinoma	2015	159	75	47.20%	IHC	using a 4-value intensity score ( >2 score)
Zhang et al.	Rectal adenocarcinoma	2015	90	47	52.20%	IHC	> median expression levels
Bruno et al.	Prostate Cancer	2015	285	NA	NA	IF	> median mean fluorescence intensity
Ren et al.	HNSCC	2016	162	100	61.70%	IHC	> median expression levels
Ren et al.	Oral squamous cell carcinoma	2016	113	66	58.40%	IHC	> 10% positively stained cells
Zhang et al.	Colorectal Cancer	2016	566	283	50.00%	MA	> median expression levels
Yusuke et al.	Non-small-cell lung cancer	2017	642	66	10.30%	IHC	H-scores that met or exceeded the individual cutoffs

*4-value grade: CD73 expression levels were graded on a scale of 0 to 3 based on cytoplasmic and membrane staining intensity and the proportion of positive tumor cells by an expert pathologist who was blinded to the patient’s clinical records. The staining was graded as 0 if no cancer cells were reactive, 1 if staining was weakly positive in <1/3 of cancer cells, 2 if staining was weakly positive in >2/3 of cancer cells, or strongly positive in >1/3 of cancer cells, and 3 if staining was weakly positive in most cancer cells, or strongly positive in >2/3 of cancer cells. Immunohistochemical staining for CD73 in ovarian cancer tissue was classified as negative (grade 0) or positive (grade 1 to 3).

**Semi-quantitative method: The percentage of positive cells was scored 0 for staining of < 1%, 1 for staining of 2%-25%, 2 for staining of 26%-50%, 3 for staining of 51%-75%, and 4 for staining > 75% of the cells examined. Staining intensity was calculated, no coloring, slightly yellow, brown yellow and tan stains were marked as 0, 1, 2 and 3. Finally, we calculated the product of staining intensity and positive cell percentage: ≤ 5 was de ned as negative and ≥ 6 as positive.

***Semi-quantitative 3-scale scoring system, score 0: no staining; score 1+: weak staining; score 2+: strong staining.

H-scores were calculated by multiplying the intensity score (0, absent; 1, weak; 2, moderate; 3, strong) by the percentage of stained cells (0–100%) to yield a value of 0–300.

No., number; IHC, Immunohistochemistry; IF, immunofluoresence; MA, microarray analysis; HNSCC, Head and neck squamous cell carcinoma; NA, not applicable.

### Test method of high CD73 expression

Immunohistochemistry (IHC), immunofluorescence (IF) and microarray analysis (validated with another method) were used to test CD73 expression. IHC was the most commonly used method (12 of 14). Of note, the criteria for high CD73 expression were distinct among different studies using IHC. For example, in some studies, the percentage of positive-staining tumor cells larger than median expression level were considered to be high CD73 expression. In other studies, staining intensity > 10% of positive-staining tumor cells was taken as high CD73 expression. Semi-quantitative 3-scale scoring system and 4-value grade were commonly used criterion, which were obtained for each case by multiplying the percentage and intensity score. The definition of positive expression of CD73 were summarized in Table 1. Nevertheless, in these studies used this scoring system, the cutoff points were distinct among different studies.

### Prevalence of high CD73 expression

**Fig. 2:**
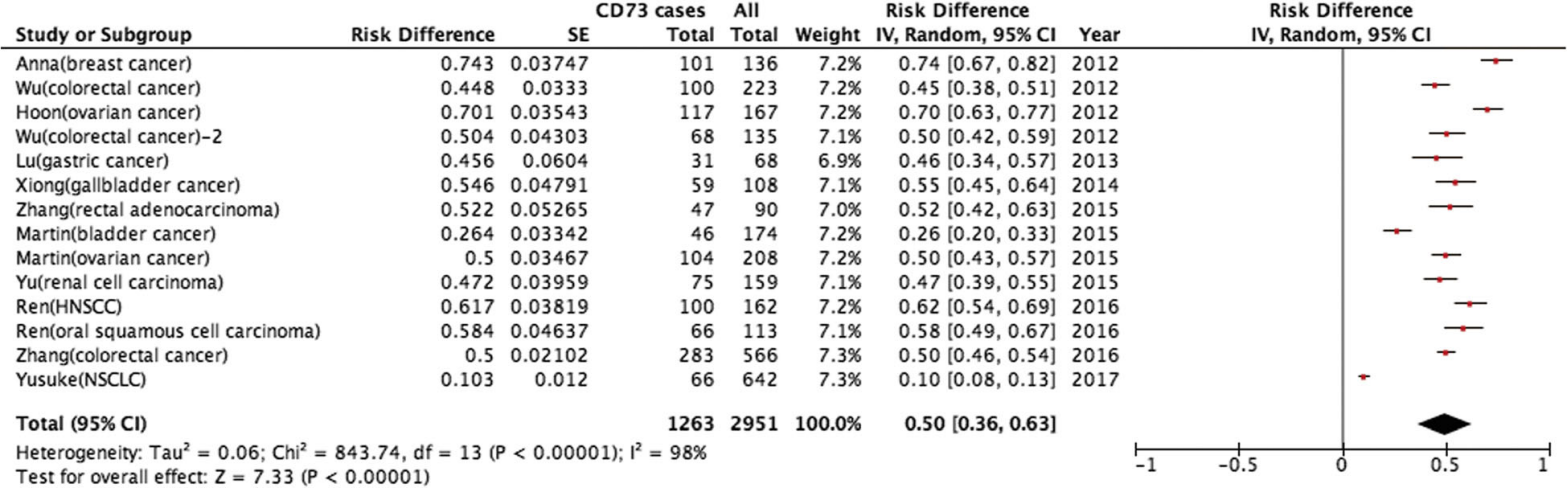
Meta-analysis of the prevalence of CD73 overexpression in all included studies

The incidence of high CD73 expression in these studies ranged from 10.30% to 74.30%, partly reflecting the heterogeneity in the criteria for high expression. In the meta-analysis of 14 studies, the incidence of high CD73 expression was 0.50 (95% CI: 0.36–0.63) and large heterogeneity existed (I^2^ = 98.0%; *P* < 0.05; Fig. 2). Subgroup analysis was stratified by test methods (IHC) and evaluation criteria, but the heterogeneity could not be reduced.

The expression level of CD73 in different cancers were explored by using the data from Oncomine. As shown in Supplemental Material, median CD73 expression level in tumor tissues was significantly higher than that in normal tissues in most kinds of cancers including bladder, brain, invasive lobular breast, esophageal, gastric, pancreatic cancer, rectal mucinous, renal cell, lung large cell, oral cavity squamous cell carcinoma, melanoma, and lung adenocarcinoma (*P* < 0.05) (Additional file 1: Figures S1, S2, S3, S5, S6, S7, S8, S11, S12, S13, S15, S17). However, several types of tumors (cervical, liver, colorectal, prostate invasive ductal breast, small cell lung cancer and lung squamous cell carcinoma) showed similar CD73 expression level compared to the level in matched normal tissues (*P* > 0.05) (Additional file 1: Figures S3, S4, S5, S9, S10, S11, S12, S14, S18, S19). Notably, CD73 expression in cecum adenocarcinoma or ovarian cancer was markedly lower than that in matched normal tissue (*P* < 0.05) (Additional file 1: Figures S5, S16). CD73 expression level in different histological types of one cancer was heterogeneous. For example, invasive lobular breast cancer has the higher CD73 expression level while invasive ductal breast cancer has the lower CD73 expression level (Additional file 1: Figure S3). In lung cancer, histology of large cell carcinoma has the significantly higher CD73 expression level but histology of small cell lung cancer and squamous cell carcinoma has the markedly lower expression level than that in matched normal tissue (Additional file 1: Figure S11).

### Relationship between high CD73 expression and prognosis

**Fig. 3:**
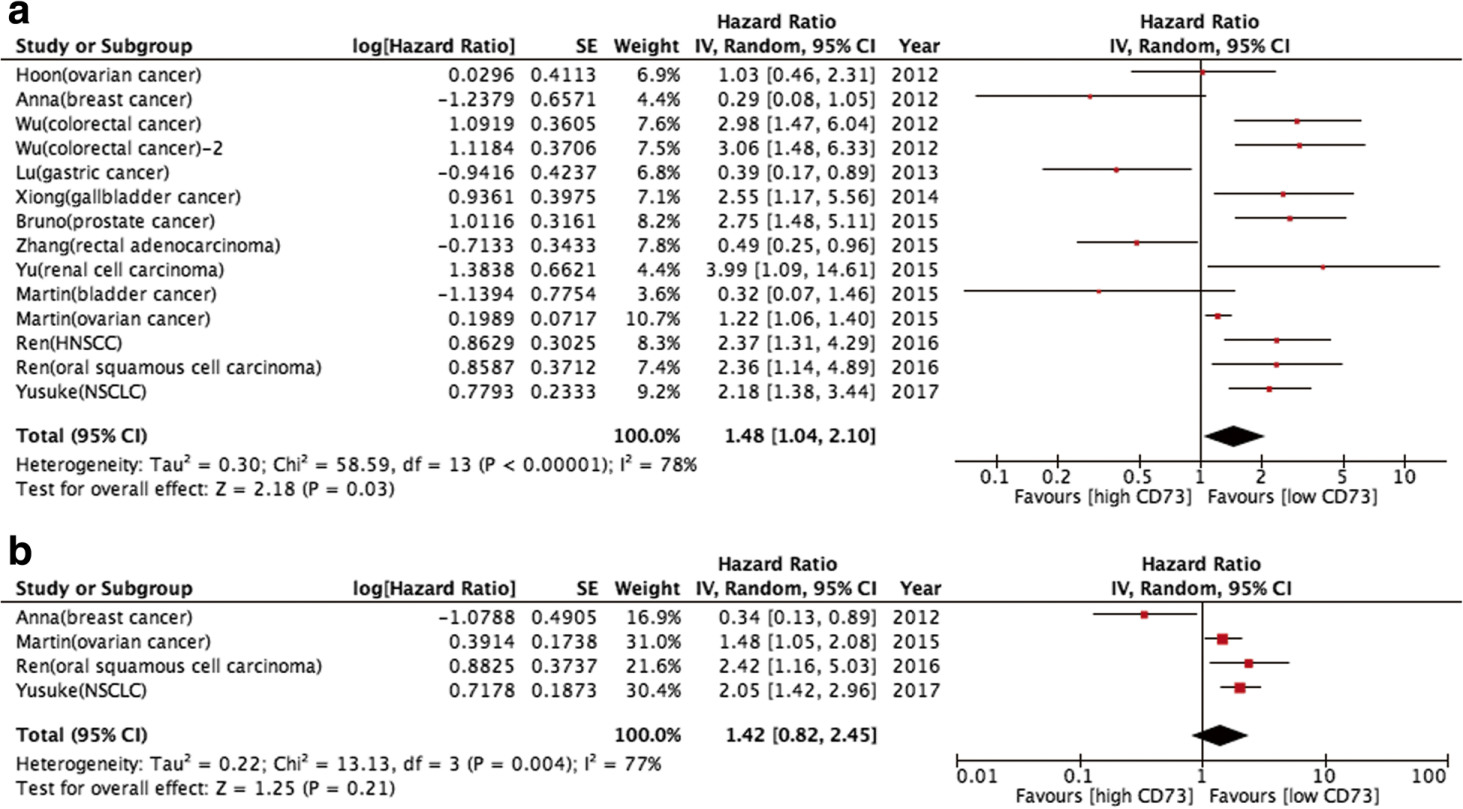
Prognostic value of CD73 overexpression in patients with cancer. **a** meta-analysis of CD73 overexpression and overall survival in various cancers; (**b**) meta-analysis of CD73 overexpression and recurrence free survival in various cancers

Pooled analysis was used to assess high CD73 expression overall effect for the studies containing prognostic data. The results showed that high CD73 expression was significantly correlated with poorer OS in various cancers [HR 1.48 (95% CI: 1.04–2.10); *P* = 0.030] but large heterogeneity existed (I^2^ = 78.0%; *P* < 0.05; Fig. 3a). In the four studies that reported RFS, the pooled result indicated that high CD73 expression was not associated with RFS [HR: 1.42 (95% CI: 0.82–2.45); *P* = 0.210; Fig. 3b]. The results also showed high heterogeneity (I^2^ = 77.0%; *P* < 0.05).

**Fig. 4:**
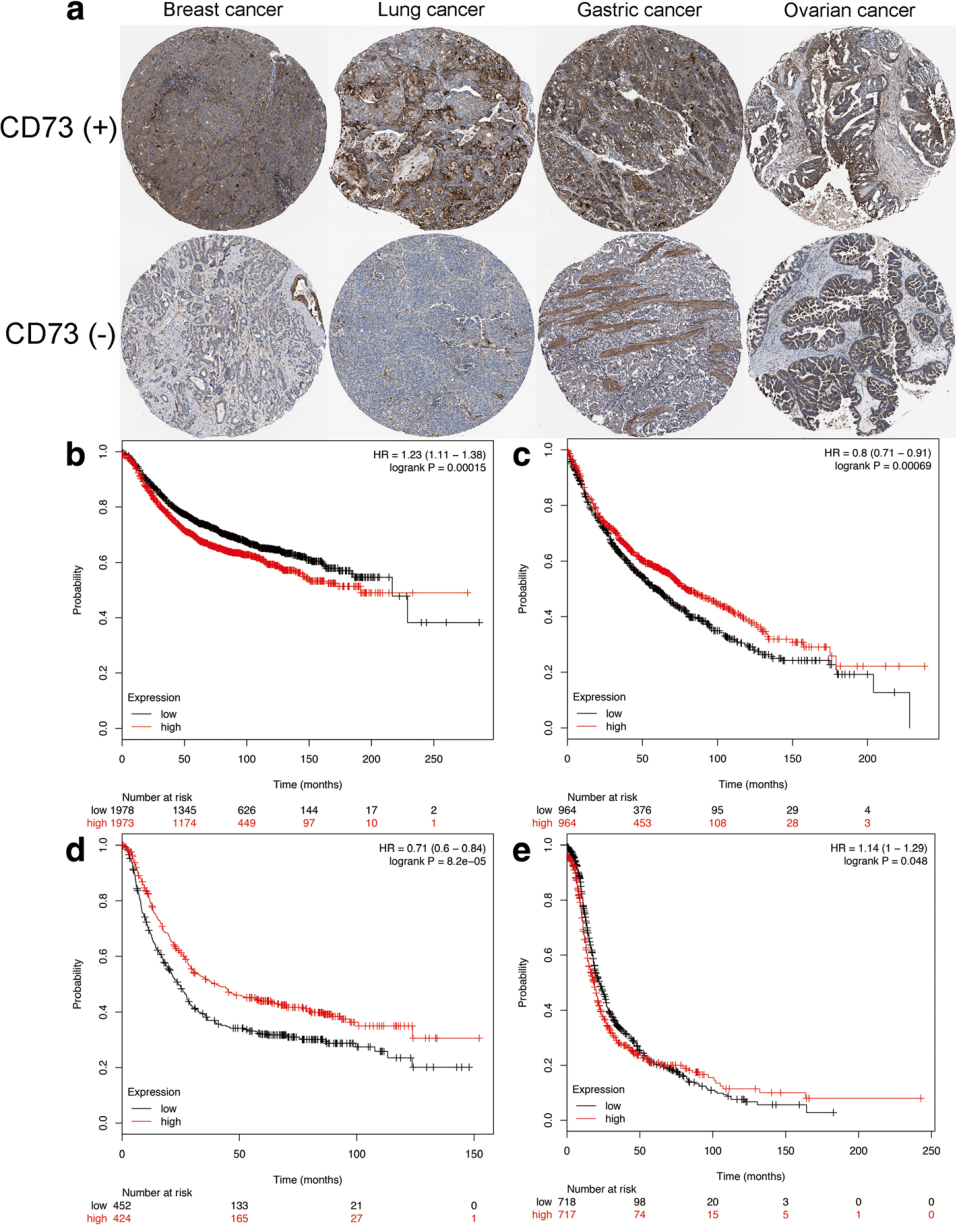
The association between CD73 overexpression and prognosis in breast, lung, gastric and ovarian cancer based on the published data. **a** The representative figures of CD73 overexpression and negative CD73 expression in breast, lung and gastric and ovarian cancer were obtained from the Human Protein Atlas; (**b**, **c**, **d**, **e**) association between CD73 overexpression and prognosis in breast, lung, gastric and ovarian cancer

In addition, we carried out the subgroup analysis of association between high CD73 expression and prognosis in breast, lung, gastric and ovarian cancer by using online database. The representative figures of high CD73 expression and negative CD73 expression in breast, lung and gastric and ovarian cancer were obtained from the Human Protein Atlas (proteinatlas.org) with the approval and listed in Fig. 4a. Consistent with the meta-analysis, the results from database showed that high CD73 expression was significantly correlated with poor OS in breast [HR: 1.23 (95% CI: 1.11–1.38); *P* < 0.05; Fig. 4b] and ovarian cancer [HR: 1.14 (95% CI: 1.00–1.29); *P* < 0.05; Fig. 4e]. However, high CD73 expression was correlated with favorable OS in lung [HR: 0.80 (95% CI: 0.71–0.91); *P* < 0.05; Fig. 4c] and gastric cancer [HR: 0.71 (95% CI: 0.60–0.84); *P* < 0.05; Fig. 4d].

### Relationship between high CD73 expression and clinicopathological parameters

**Fig. 5:**
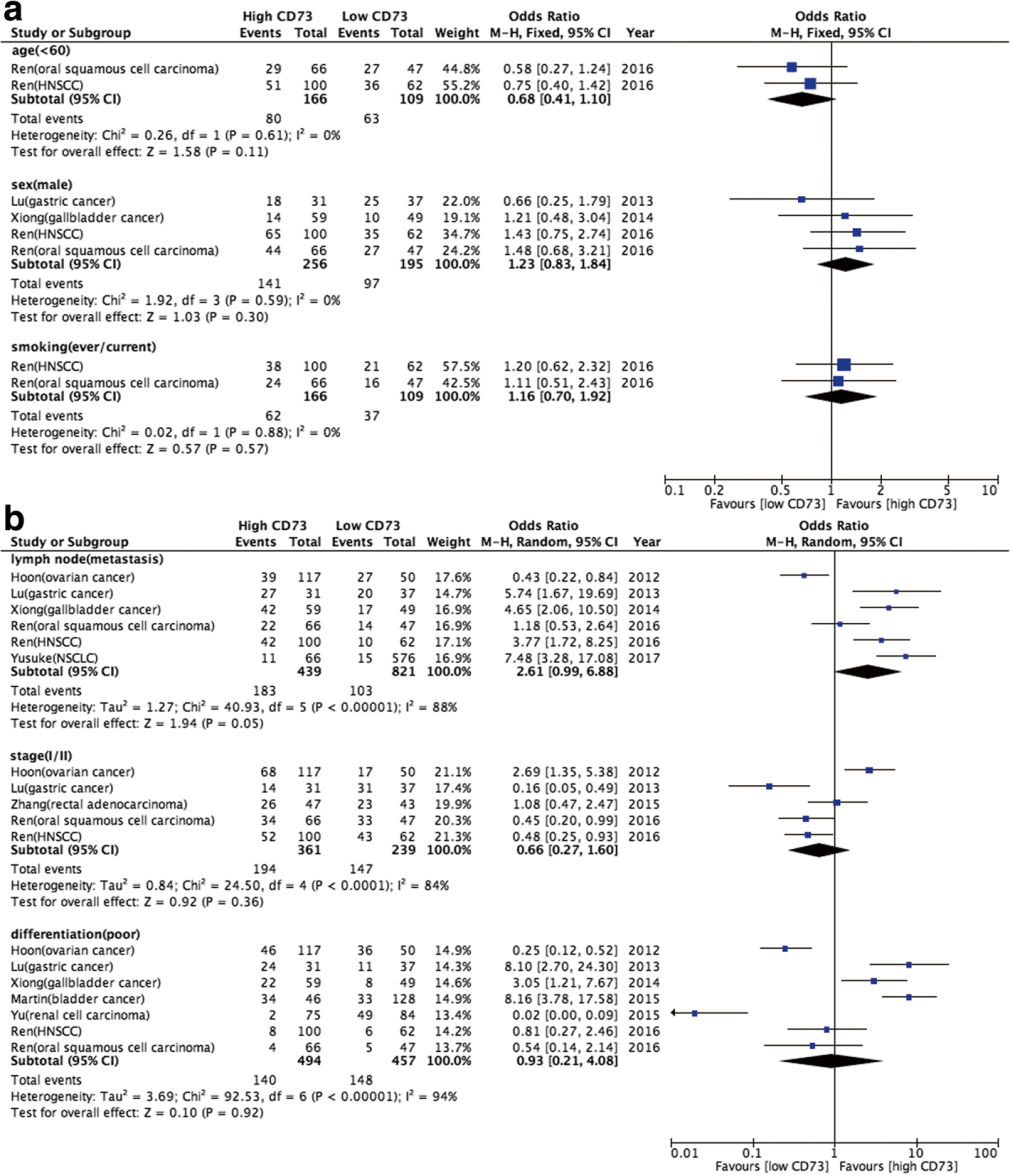
The relationship between clinicopathological features and CD73 overexpression in different cancers. **a** meta-analysis of CD73 overexpression and age < 60 years-old, male gender and smoking history; (**b**) meta-analysis of CD73 overexpression and lymph node metastasis, clinical stage and tumor differentiation

To investigate the relationship between high CD73 expression and clinicopathological features, meta-analyses were performed according to the different characteristics. As the results suggested, high CD73 expression was dramatically associated with lymph node metastasis [OR: 2.61 (95CI: 0.99–6.88); *P* = 0.05] but high CD73 expression was not correlated with the other reported clinicopathological features including age, gender, smoking history, clinical stage and differentiation (Fig. 5).

### Sensitivity and publication bias

Sensitivity analysis was conducted by deleting one study at one time to assess its effect on prevalence and pooled HRs. Deletion of the study by Martin et al. [21] and Yusuke et al. [26] slightly decreased the heterogeneity in the analysis of high CD73 expression prevalence. No other individual study influenced the results. Begg’s funnel plots and Egger’s tests evaluated the publication bias, and it was only detected in the analysis of high CD73 expression prevalence (*P* < 0.05 for Egger’s test). Further analyses showed that the Begg’s funnel plot was symmetric and Egger’s tests suggested that there was no evidence of publication bias (Additional file 1: Figure S20).

## Discussion

To our knowledge, the current study is the one of the first studies to comprehensively investigate the characterization of CD73 and its effect on prognosis in various solid tumors. In the current study, the pooled results showed that CD73 highly expressed in 12 types of human cancers and the prevalence of high CD73 expression was more than 50%. Due to the limited number of included cases, we assessed the expression level of CD73 in a broader set of cancers versus matched normal tissues by using the web-based microarray database (Oncomine). The results further suggested that CD73 highly expressed in most kinds of cancers including bladder, brain, invasive lobular breast, esophageal, gastric, pancreatic cancer, rectal mucinous, renal cell, lung large cell, oral cavity squamous cell carcinoma, melanoma, and lung adenocarcinoma. However, several types of tumors (cervical, liver, colorectal, prostate invasive ductal breast, small cell lung cancer and lung squamous cell carcinoma) showed similar CD73 expression level compared to that in matched normal tissues. Notably, cecum adenocarcinoma or ovarian cancer had the lower CD73 expression level than that in matched normal tissue. Consistently, Lu et al. reported that high CD73 expression was found in 45.60% of patients with gastric cancer [11]. Yu and colleagues also found that 47.20% of renal cell carcinoma highly expressed CD73 [22]. Of note, Hoon et al. collected 167 patients with epithelial ovarian cancer and found that 70.1% of patients showed positive expression for CD73 while data from Oncomine showed that ovarian cancer had low CD73 expression level. The reason for this discrepancy may include that the histological type of included ovarian cancer and test methods are different. Histology of ovarian cancer in Oncomine were mucinous, serous, endometrioid and clear cell adenocarcinoma and CD73 expression were analyzed based on microarray analysis whereas Hoon’ study enrolled epithelial ovarian carcinoma and used IHC to assess the expression of CD73. These results indicated that distinct histological types of cancers would have distinct CD73 expression.

Furthermore, we investigated the relationship between high CD73 expression and prognosis in different cancers. The results of all included studies demonstrated that high CD73 expression was significantly associated with poor OS but not RFS. In virtue of the high heterogeneity and small number of included studies, we performed the validation subgroup analysis via “The Kaplan-Meier plotter” (KM plotter) database which is capable to assess the effect of 54,675 genes on survival of patients with breast, lung, gastric and ovarian cancer [27]. The pooled results showed that high CD73 expression was markedly associated with poor OS in breast and ovarian cancer but favorable OS in lung and gastric cancer. In breast cancer, a previous study demonstrated that positive CD73 expression was correlated with longer DFS and OS, which was opposite to the results from KM plotter. Theoretically, cancer cells with high CD73 expression possessed higher aggressiveness and invasiveness [28]. Leth-Larsen and colleagues also showed that intense NT5E/CD73 IHC staining was more common for breast cancer patients with relapse and lymph node metastases [29]. Hence, it seems that high CD73 expression was likely to be associated with poor prognosis in breast cancer. In lung cancer, however, Yusuke et al. also reported the contrary results that high CD73 expression was an independent indicator of poor prognosis for OS and RFS. The reason underlying this discrepancy is unclear as there are few studies to deeply explore the functions of CD73 in immune cells and tumor microenvironment of lung cancer [3]. The complex signaling pathway downstream to CD73-adenosince in lung cancer cells should be investigated to provide a preciously mechanistic explanation [26]. Of note, data on CD73 expression from KM plotter was on the basis of gene expression data whereas the published articles utilized the IHC to assess CD73 expression level. As is known, gene expression level is not positively related to the corresponding protein expression level. Moreover, the cutoff value of positive CD73 expression is also different. These would result in the difference on the association between CD73 expression and prognosis.

As to the clinicopathological characteristics, we found that high CD73 expression was significantly associated with lymph node metastasis but not correlated with the other reported clinicopathological features including age, gender, smoking history, clinical stage and differentiation. Consistently, Lu et al. enrolled 68 patients with resected gastric carcinoma and found that overexpression of CD73 was positively associated with lymph node metastases (*P* = 0.003) [11]. Similar result in gallbladder cancer was reported by Xiong and colleagues [19]. Furthermore, Ren et al. collected 162 patients with head and neck squamous cell carcinoma (HNSCC) and highlighted that there was a direct relationship between CD73 expression and lymph node metastases (*P* < 0.001). They further demonstrated that CD73 could promote HNSCC migration and invasion via adenosine A3R stimulation and the activation of EGF/EGFR signaling [24]. This could be one of the potential mechanism for the close relationship between CD73 expression and lymph node metastases.

Accumulating evidence indicates that CD73-adenosine pathway plays a crucial role in cancer progression and immune escape. A series of studies suggested that CD73-derived adenosine could help to form immunosuppressive environment via dampening anti-tumor effect of immune cells, such as CD8+ positive T cells and NK cells [3, 30]. Stagg et al. firstly reported that targeted blockade of CD73 could reduce the tumor growth and metastasis in immune-competent mice through the activation of adaptive anti-tumor immunity [31]. After that, emerging evidence highlights the critical role of CD73 in the regulation of MDSC expansion, M2 macrophages polarization and Treg inhibitory activity [4, 32–34]. Recently, several studies showed that CD73 expression on tumor cells weakened the immune response to PD-1/PD-L1 inhibitors [35, 36]. Allard et al. reported that anti-CD73 mono-antibody (mAb) dramatically enhanced the effect of anti-CTLA-4 and PD-1 inhibitors against colon, prostate and breast cancers in mice model [36]. Iannone and colleagues also found that blockade of CD73 could enhance efficacy of anti-CTLA-4 in melanoma model [37]. Beavis et al. further showed that combination of CD73-A2A inhibition and anti-PD-1 mAb resulted in greater antitumor immune response through prolonged expression of IFN-gamma and granzyme B [35]. These results suggested that CD73 was a potential biomarker for response to anti-PD-1/PD-L1 treatment.

Targeting CD73 also showed the favorable antitumor effects in preclinical studies [38, 39]. To date, several potent inhibitors or antibodies of CD73 have been discovered via high-throughput drug screenings. One of the most valuable drugs is MEDI9447. MEDI9447 could enhance the activity of PD-1 antibody in a syngeneic tumor model through increasing CD8+ T cells and reducing MDSC and Tregs in the tumor microenvironment [40, 41]. Herein, they performed a phase I clinical trial of the MEDI9447 in patients with advanced solid tumors, as single agent and in combination with the anti-PD-L1 antibody (NCT02503774). Another small molecular inhibitor, PBF-509, is the A2A receptor antagonist. A phase I study on PBF-509 in immunotherapy-naïve, locally advanced or metastatic NSCLC patients is ongoing (NCT02403193).

There are several limitations of our study. Firstly, the publication bias was inevitable. Several abstracts were identified but not further detailed in standard publications. Although we have tried our best to contact authors of primary studies, no reply was received. Therefore, we could not include these data. Secondly, the quality of data on the incidence of high CD73 expression was statistically heterogeneous among the studies. Thirdly, it is difficult to make a direct comparison between distinct studies due to several confounding factors including lab condition, test techniques and platform, definition of positive CD73 expression and so on.

## Conclusions

In summary, the current study indicates that high CD73 expression would be a potential prognostic factor to human solid tumors, especially the lung, breast, gastric and ovarian cancer. High CD73 expression was correlated with distant/local lymph node metastases. CD73 is also a promising target in future cancer immunotherapy and has the potential significance as a biomarker for anti-PD-1/PD-L1 treatment.

## Additional file

Additional file 1: Figure S1. The expression level of CD73/NT5E in bladder cancer versus matched normal tissue. **Figure S2**. The expression level of CD73/NT5E in brain cancer versus matched normal tissue. **Figure S3**. The expression level of CD73/NT5E in breast cancer versus matched normal tissue. **Figure S4**. The expression level of CD73/NT5E in cervical cancer versus matched normal tissue. **Figure S5**. The expression level of CD73/NT5E in colorectal cancer versus matched normal tissue. **Figure S6**. The expression level of CD73/NT5E in esophageal cancer versus matched normal tissue. **Figure S7**. The expression level of CD73/NT5E in gastric cancer versus matched normal tissue. **Figure S8**. The expression level of CD73/NT5E in kidney cancer versus matched normal tissue. **Figure S9**. The expression level of CD73/NT5E in leukemia versus matched bone marrow. **Figure S10**. The expression level of CD73/NT5E in liver cancer versus matched normal tissue. **Figure S11**. The expression level of CD73/NT5E in lung cancer versus matched normal tissue. **Figure S12**. The expression level of CD73/NT5E in lymphoma versus matched CD4+ T lymphocyte. **Figure S13**. The expression level of CD73/NT5E in melanoma versus matched normal tissue. **Figure S14**. The expression level of CD73/NT5E in myeloma versus matched plasma cell. **Figure S15**. The expression level of CD73/NT5E in oral cavity squamous cell carcinoma versus matched normal tissue. **Figure S16**. The expression level of CD73/NT5E in ovarian cancer versus matched normal tissue. **Figure S17**. The expression level of CD73/NT5E in pancreatic cancer versus matched normal tissue. **Figure S18**. The expression level of CD73/NT5E in prostate cancer versus matched normal tissue. **Figure S19**. The expression level of CD73/NT5E in sarcoma versus matched normal tissue. **Figure S20**. Publication bias for the prevalence of CD73/NT5E in various cancers. (DOCX 721 kb)

## Abbreviations

AMP: Adenosine monophosphate
CI: Confidence interval
CTLA-4: Cytotoxic T-lymphocyte antigen-4
GPCR: G-protein coupled receptor
HNSCC: Head and neck squamous cell carcinoma
HR: high risk
IF: Immunofluorescence
IHC: Immunohistochemistry
mAb: Mono-antibody
NK: Natural killer
NSCLC: Non-small-cell lung cancer
NT5E: ecto-5′-nucleotidase
OS: Overall survival
PD-1: Programmed cell death protein-1
RFS: recurrence free survival
