# Supplementary material for: Comprehensive evaluation of NT5E/CD73 expression and its prognostic significance in distinct types of cancers
Source: BMC Cancer. 2018 Mar 7;18:267. doi: 10.1186/s12885-018-4073-7 (PMC5842577; doi:10.1186/s12885-018-4073-7)
Supplement: Supplementary file 1 — The expression level of CD73/NT5E in bladder cancer versus matched normal tissue. Figure S2. The expression level of CD73/NT5E in brain cancer versus matched normal tissue. Figure S3. The expression level of CD73/NT5E in breast cancer versus matched normal tissue. Figure S4. The expression level of CD73/NT5E in cervical cancer versus matched normal tissue. Figure S5. The expression level of CD73/NT5E in colorectal cancer versus matched normal tissue. Figure S6. The expression level of CD73/NT5E in esophageal cancer versus matched normal tissue. Figure S7. The expression level of CD73/NT5E in gastric cancer versus matched normal tissue. Figure S8. The expression level of CD73/NT5E in kidney cancer versus matched normal tissue. Figure S9. The expression level of CD73/NT5E in leukemia versus matched bone marrow. Figure S10. The expression level of CD73/NT5E in liver cancer versus matched normal tissue. Figure S11. The expression level of CD73/NT5E in lung cancer versus matched normal tissue. Figure S12. The expression level of CD73/NT5E in lymphoma versus matched CD4+ T lymphocyte. Figure S13. The expression level of CD73/NT5E in melanoma versus matched normal tissue. Figure S14. The expression level of CD73/NT5E in myeloma versus matched plasma cell. Figure S15. The expression level of CD73/NT5E in oral cavity squamous cell carcinoma versus matched normal tissue. Figure S16. The expression level of CD73/NT5E in ovarian cancer versus matched normal tissue. Figure S17. The expression level of CD73/NT5E in pancreatic cancer versus matched normal tissue. Figure S18. The expression level of CD73/NT5E in prostate cancer versus matched normal tissue. Figure S19. The expression level of CD73/NT5E in sarcoma versus matched normal tissue. Figure S20. Publication bias for the prevalence of CD73/NT5E in various cancers. (DOCX 721 kb) [file 12885_2018_4073_MOESM1_ESM.docx]

**Supplementary Material**

**Comprehensive evaluation of NT5E/CD73 expression and its prognostic significance in distinct types of cancers**

Tao Jiang, Xiaofeng Xu, Meng Qiao, Xuefei Li, Chao Zhao, Fei Zhou, Guanghui Gao, Fengying Wu, Xiaoxia Chen, Chunxia Su, Shengxiang Ren, Caicun Zhou

**SUPPLEMENTAL FIGURES**

**Supplementary Figure S1.** The expression level of CD73/NT5E in bladder cancer versus matched normal tissue.

**Supplementary Figure S2.** The expression level of CD73/NT5E in brain cancer versus matched normal tissue.

**Supplementary Figure S3.** The expression level of CD73/NT5E in breast cancer versus matched normal tissue.

**Supplementary Figure S4.** The expression level of CD73/NT5E in cervical cancer versus matched normal tissue.

**Supplementary Figure S5.** The expression level of CD73/NT5E in colorectal cancer versus matched normal tissue.

**Supplementary Figure S6.** The expression level of CD73/NT5E in esophageal cancer versus matched normal tissue.

**Supplementary Figure S7.** The expression level of CD73/NT5E in gastric cancer versus matched normal tissue.

**Supplementary Figure S8.** The expression level of CD73/NT5E in kidney cancer versus matched normal tissue.

**Supplementary Figure S9.** The expression level of CD73/NT5E in leukemia versus matched bone marrow.

**Supplementary Figure S10.** The expression level of CD73/NT5E in liver cancer versus matched normal tissue.

**Supplementary Figure S11.** The expression level of CD73/NT5E in lung cancer versus matched normal tissue.

**Supplementary Figure S12.** The expression level of CD73/NT5E in lymphoma versus matched CD4+ T lymphocyte.

**Supplementary Figure S13.** The expression level of CD73/NT5E in melanoma versus matched normal tissue.

**Supplementary Figure S14.** The expression level of CD73/NT5E in myeloma versus matched plasma cell.

**Supplementary Figure S15.** The expression level of CD73/NT5E in oral cavity squamous cell carcinoma versus matched normal tissue.

**Supplementary Figure S16.** The expression level of CD73/NT5E in ovarian cancer versus matched normal tissue.

**Supplementary Figure S17.** The expression level of CD73/NT5E in pancreatic cancer versus matched normal tissue.

**Supplementary Figure S18.** The expression level of CD73/NT5E in prostate cancer versus matched normal tissue.

**Supplementary Figure S19.** The expression level of CD73/NT5E in sarcoma versus matched normal tissue.

**Supplementary Figure S20.** Publication bias for the prevalence of CD73/NT5E in various cancers.

**Supplementary Figure S1**


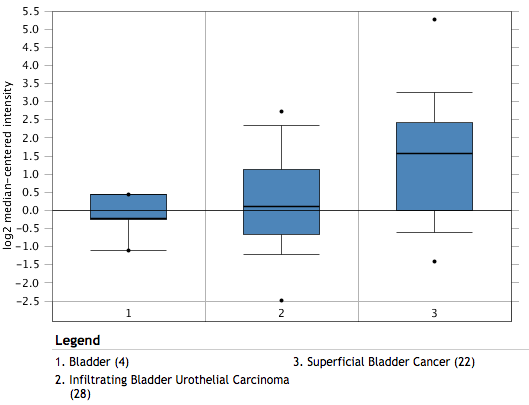


**Supplementary Figure S1.** The expression level of CD73/NT5E in bladder cancer versus matched normal tissue.

**Supplementary Figure S2**


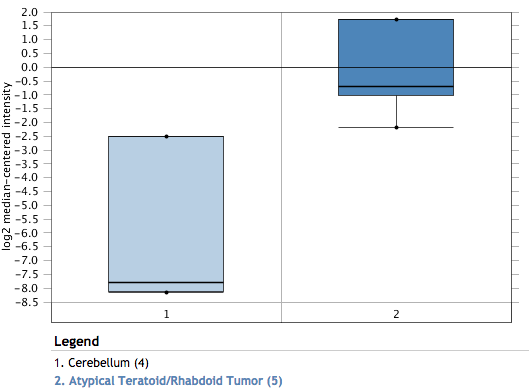


**Supplementary Figure S2.** The expression level of CD73/NT5E in brain cancer versus matched normal tissue.

**Supplementary Figure S3**


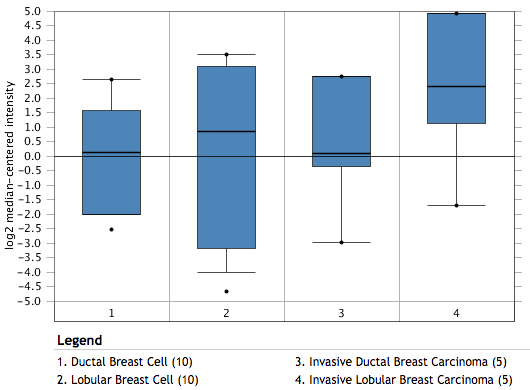


**Supplementary Figure S3.** The expression level of CD73/NT5E in breast cancer versus matched normal tissue.

**Supplementary Figure S4**


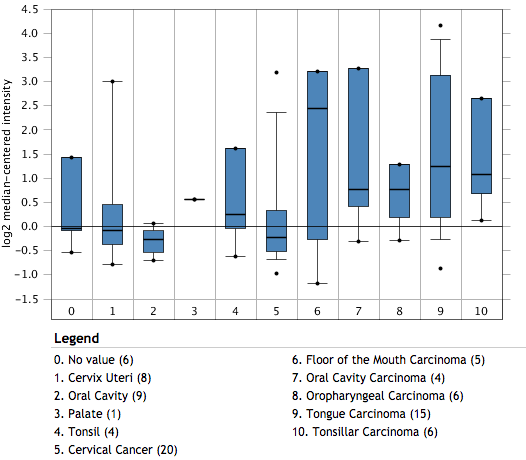


**Supplementary Figure S4.** The expression level of CD73/NT5E in cervical cancer versus matched normal tissue.

**Supplementary Figure S5**


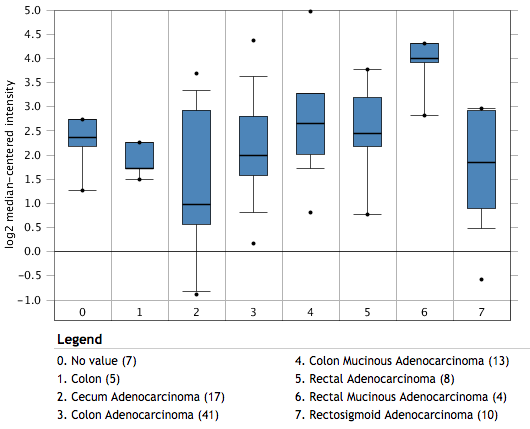


**Supplementary Figure S5.** The expression level of CD73/NT5E in colorectal cancer versus matched normal tissue.

**Supplementary Figure S6**


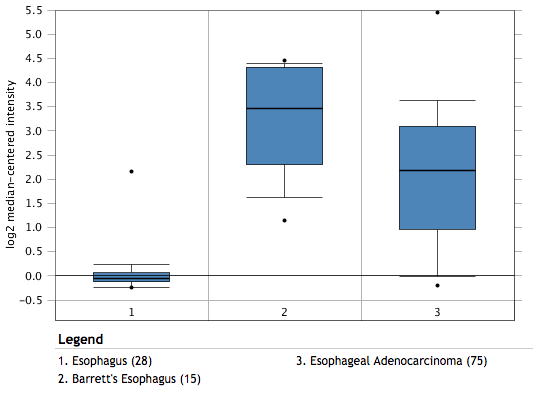


**Supplementary Figure S6.** The expression level of CD73/NT5E in esophageal cancer versus matched normal tissue.

**Supplementary Figure S7**


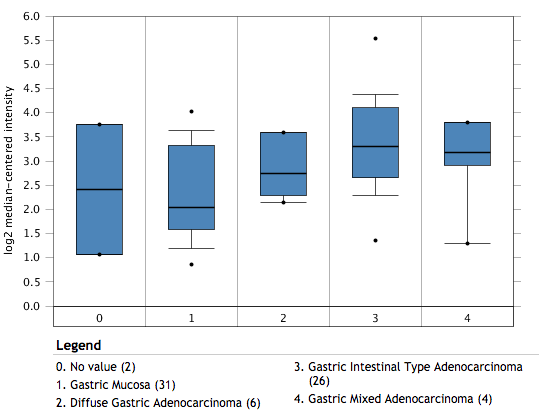


**Supplementary Figure S7.** The expression level of CD73/NT5E in gastric cancer versus matched normal tissue.

**Supplementary Figure S8**


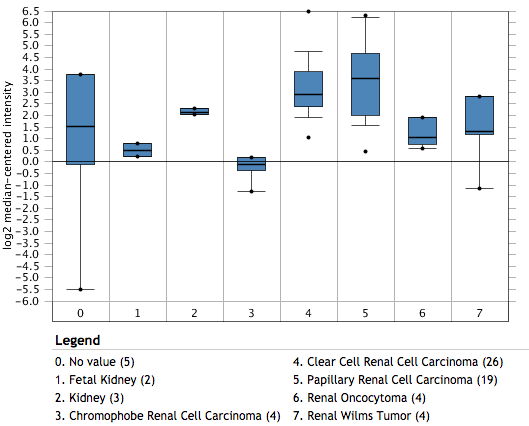


**Supplementary Figure S8.** The expression level of CD73/NT5E in kidney cancer versus matched normal tissue.

**Supplementary Figure S9**


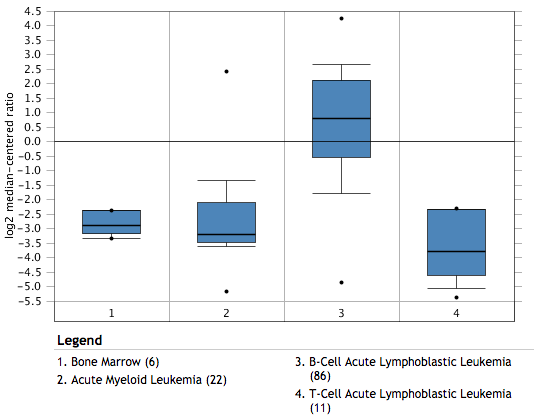


**Supplementary Figure S9.** The expression level of CD73/NT5E in leukemia versus matched bone marrow.

**Supplementary Figure S10**


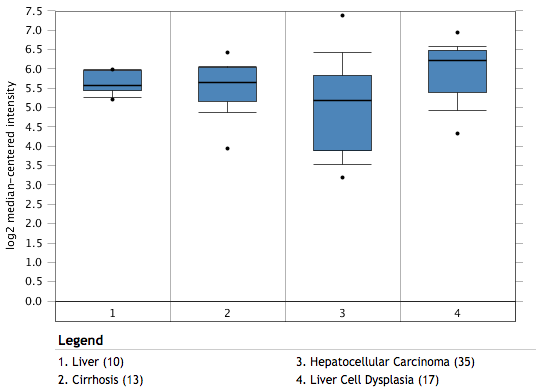


**Supplementary Figure S10.** The expression level of CD73/NT5E in liver cancer versus matched normal tissue.

**Supplementary Figure S11**


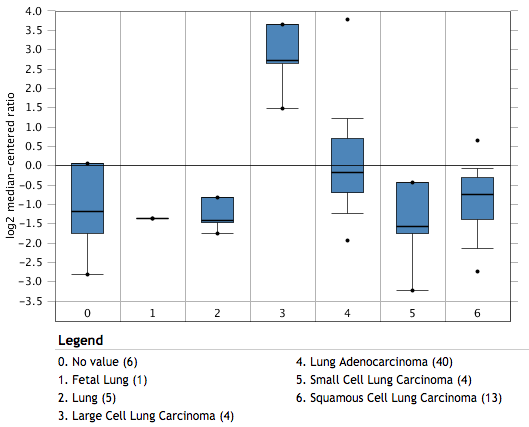


**Supplementary Figure S11.** The expression level of CD73/NT5E in lung cancer versus matched normal tissue.

**Supplementary Figure S12**


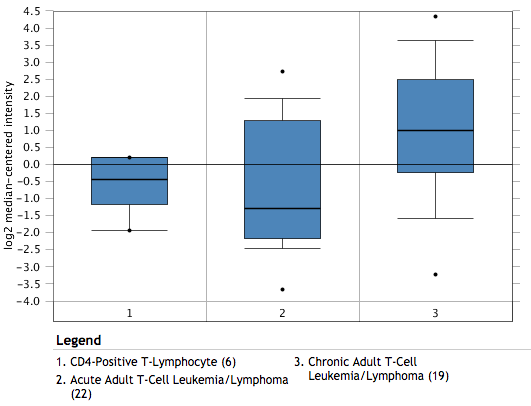


**Supplementary Figure S12.** The expression level of CD73/NT5E in lymphoma versus matched CD4+ T lymphocyte.

**Supplementary Figure S13**


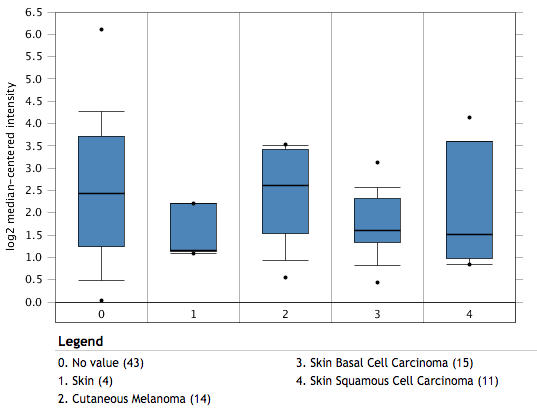


**Supplementary Figure S13.** The expression level of CD73/NT5E in melanoma versus matched normal tissue.

**Supplementary Figure S14**


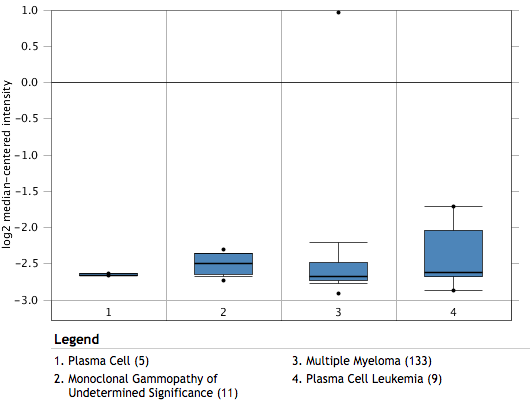


**Supplementary Figure S14.** The expression level of CD73/NT5E in myeloma versus matched plasma cell.

**Supplementary Figure S15**


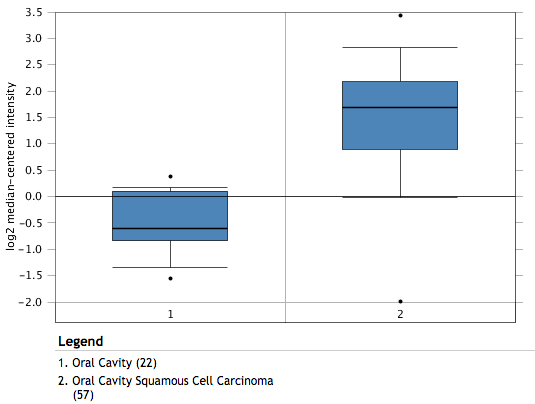


**Supplementary Figure S15.** The expression level of CD73/NT5E in oral cavity squamous cell carcinoma versus matched normal tissue.

**Supplementary Figure S16**


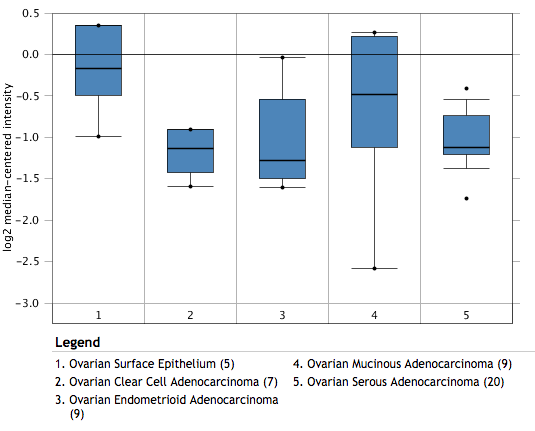


**Supplementary Figure S16.** The expression level of CD73/NT5E in ovarian cancer versus matched normal tissue.

**Supplementary Figure S17**


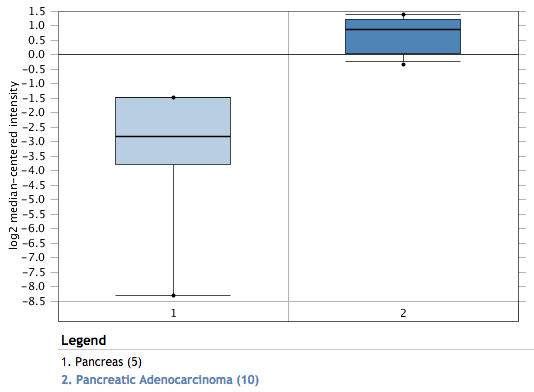


**Supplementary Figure S17.** The expression level of CD73/NT5E in pancreatic cancer versus matched normal tissue.

**Supplementary Figure S18**


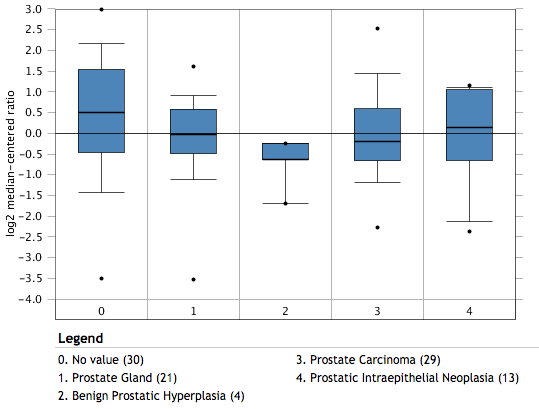


**Supplementary Figure S18.** The expression level of CD73/NT5E in prostate cancer versus matched normal tissue.

**Supplementary Figure S19**


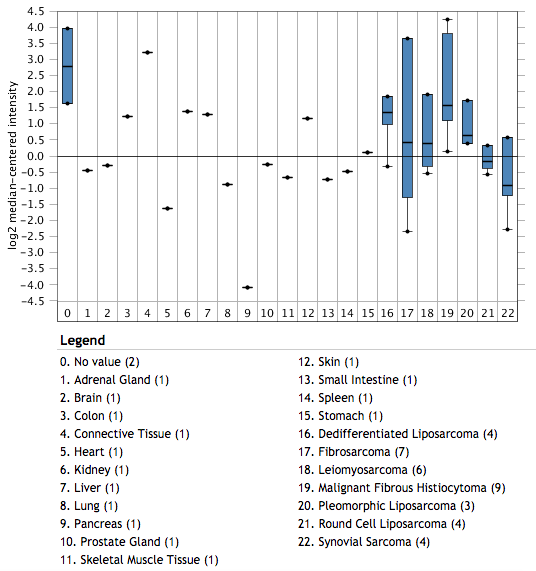


**Supplementary Figure S19.** The expression level of CD73/NT5E in sarcoma versus matched normal tissue.

**Supplementary Figure S20**


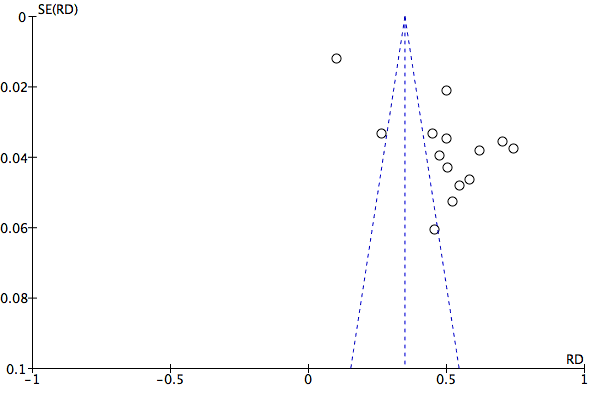


**Supplementary Figure S20.** Publication bias for the prevalence of CD73/NT5E in various cancers.
